# The prognostic value of standardized phase angle in adults with acute leukemia: A prospective study

**DOI:** 10.1002/cam4.2835

**Published:** 2020-02-12

**Authors:** Samuel J. Yates, Susan Lyerly, Megan Manuel, Janet A. Tooze, Heidi D. Klepin, Bayard L. Powell, Sarah Dralle, Alok Uprety, Timothy S. Pardee

**Affiliations:** ^1^ Wake Forest School of Medicine Winston‐Salem NC USA; ^2^ Comprehensive Cancer Center of Wake Forest University Winston‐Salem NC USA; ^3^ Division of Public Health Sciences Wake Forest School of Medicine Winston‐Salem NC USA; ^4^ Present address: John H. Stroger, Jr. Hospital of Cook County Chicago IL USA

**Keywords:** body composition, leukemia, nutrition, phase angle, prognostic

## Abstract

Standardized phase angle (SPhA) is a tool used to estimate body composition and cell membrane integrity. Standardized phase angle has been shown to predict survival in solid malignancies and hematopoietic stem cell transplant patients. We investigated the predictive value of SPhA on 60‐day mortality, overall survival (OS), and length of hospital stay (LHS) for adults with acute myelogenous and lymphoblastic leukemia (AML and ALL). Consecutive patients ≥18 years with newly diagnosed acute leukemia receiving intensive chemotherapy were enrolled. Phase angle measurements were taken on day 1 of therapy for all patients and on the day of nadir marrow for AML patients. Measurements were standardized by BMI, gender, and age to calculate the SPhA. The difference between SPhA at nadir bone marrow compared to day 1 of induction was used to calculate change in SPhA. A cutoff of 25th percentile was used to dichotomize baseline SPhA. Among 100 patients, 88% were AML, 56% were female, and mean age was 59 years. Though not statistically significant, OS by Kaplan‐Meier analysis was shorter for those below the 25th percentile SPhA compared to those above (median OS: 11.0 months vs 19.5 months; *P* = .09). Lower baseline SPhA was associated with increased incidence of 60‐day mortality in univariable (odds ratio [OR] = 5.25; 1.35, 20.44; *P* = .02) but not multivariable analysis (OR = 3.12; 0.67, 14.48; *P* = .15) adjusted for age, creatinine, and cytogenetics. Increased change in SPhA was associated with worse OS (hazard ratio = 1.15; 1.00,1.33; *P* = .05) in multivariable analysis. Standardized phase angle is a rapid, noninvasive, and objective measure that may be used to inform risk stratification.

## Graphical abstract

Standardized phase angle (SPhA) is a tool used to estimate body composition and cell membrane integrity. Lower baseline SPhA was associated with increased incidence of 60‐day mortality in univariable (odds ratio [OR] = 5.25; 1.35, 20.44; *P* = .02) but not multivariable analysis (OR = 3.12; 0.67, 14.48; *P* = .15) adjusted for age, creatinine, and cytogenetics. Standardized phase angle is a rapid, noninvasive, and objective measure that may be used to inform risk stratification in adult acute leukemia patients.

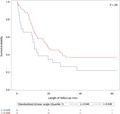

## 1 INTRODUCTION

The acute leukemias in adulthood are comprised of acute myeloid leukemia (AML) and acute lymphoblastic leukemia (ALL), a group of aggressive, molecularly heterogeneous malignancies. AML primarily affects older adults (≥60 years) with a median age at diagnosis of 67 years.^1^ Due to its peak incidence at 1‐4 years old, ALL is often perceived as a malignancy of children. However, there is another gradual increase in incidence in adults starting at 45 years of age and continuing into the older adult population.^1^ Unlike the pediatric population where cure rates approach 90%, ALL in adults has 5‐year survival rates for those 40‐75+ years old between 9.1% and 38.9%.^1^, ^2^, ^3^ Similarly, the 5‐year survival rates for AML patients 40‐64 years and ≥65 years are 37.9% and 7.1%, respectively.^1^ Furthermore, due to the increased treatment‐related mortality (TRM), there remains disagreement on whether patients should receive intensive chemotherapy, or low‐intensity chemotherapy or best supportive care.

Much work has been done to better discriminate between patients who should receive intensive chemotherapy and those who are vulnerable to increased toxicity. Tumor‐specific factors such as cytogenetics and gene mutations and patient‐specific factors, primarily age and performance status, have been used to create prognostic scoring systems for TRM and overall survival (OS).^4^, ^5^, ^6^, ^7^ Each of these variables has their limitations. Tumor‐specific factors may take >1 week to become available.^8^, ^9^, ^10^ Performance status scales lack sensitivity and are highly subjective.^11^, ^12^, ^13^ Age is primarily a surrogate marker for impairments in nutritional status, cognitive function, physical function, and psychological state.^11^ The complete geriatric assessment (CGA) has been used in ALL and AML patients in order to better characterize differences between patients of the same age.^3^, ^13^, ^14^, ^15^ However, to this point no CGA tool incorporates nutritional status assessment, a known prognostic factor in AML and ALL patients of all ages.^16^, ^17^, ^18^ Furthermore, no nutritional assessment tool (such as body mass index [BMI], weight, or subjective global assessment [SGA] questionnaires) or any of the various serum markers (albumin, pre‐albumin, and transthyretin) are consistently used in clinical practice.^18^

Bioelectrical impedance analysis is a method used to estimate body composition and cell membrane integrity. Bioelectrical impedance analysis is a useful tool as it is noninvasive, relatively inexpensive, can be performed on nearly any patient, does not expose the patient to ionizing radiation, is painless, and has both high intra‐ and interobserver precision (coefficient of variation = 2.7%‐4.0%).^19^, ^20^ Bioimpedance devices do not directly measure body composition but instead provide indirect estimates from the measurement of impedance of body tissues to an electric current.^22^ Phase angle is positively correlated with lean body mass and body cell mass and negatively correlated to the extracellular‐to‐intracellular fluid ratio (ECW/ICW) in healthy adults. As disease‐related malnutrition is classically characterized by an increase ECW/ICW and decreased body cell mass, malnutrition (assessed by pre‐albumin, albumin, and malnutrition questionnaires [SGA and nutrition risk score in the ICU]) has been shown to be negatively correlated with phase angle.^23^, ^24^, ^25^, ^26^, ^27^ Due to the contributions of age, sex, and BMI to phase angle measurements, reference values were established and validated in order to calculate the standardized phase angle (SPhA), which controls for these variables. Standardized phase angle has proven to be a strong prognostic marker for various survival outcomes in numerous solid malignancies (lung, head and neck, pancreatic, breast, and gastrointestinal) and hematopoietic stem cell transplant (HSCT) patients.^24^, ^26^, ^28^, ^29^, ^30^

To date, no studies have evaluated the usage of phase angle technology in acute leukemia patients. This prospective observational study thus sought to establish the prognostic significance of baseline SPhA and change in SPhA for TRM and OS in newly diagnosed, adult ALL and AML patients receiving intensive chemotherapy.

## 2 MATERIALS AND METHODS

### 2.1 Study population

Between July 2013 and January 2018, we conducted a single‐institution prospective observational study where consecutive patients aged ≥18 years who were newly diagnosed with pathologically confirmed AML or ALL were enrolled. Inclusion criteria included receipt of intensive (non‐hypomethylating‐based) induction chemotherapy, inpatient status, and willing and able to provide written informed consent. Patients were excluded by the presence of a pacemaker or defibrillator (due to possible interference of the bioimpedance analyzer with patient's defibrillator or pacemaker,^20^) pregnant at time of enrollment, or any condition or abnormality which would, in the opinion of the investigator, compromise the safety of the patient. The treating physician at the time of diagnosis chose the treatment regimen before enrollment in the study and recording of phase angle measurements. This study was approved by the Institutional Review Board of Wake Forest University Baptist Hospital. All participants provided written informed consent in accordance with the Declaration of Helsinki.

### 2.2 Study design and data collection

#### 2.2.1 Standardized phase angle

We followed published procedures for phase angle (PhA) measurement collection.^20^ Measurements were taken by placing 2 electrodes on the hand (ulnar head and first joint of third digit) and 2 on the foot (medial malleolus and base of second toe) on the same side of the patient, generally the right side. Participants were supine with their arms at a 30° angle to their body and legs not touching each other or electrically conductive material of the inpatient bed. A single‐frequency (50 Hz) alternating electrical current was then applied, and reactance, capacitance, and PhA were recorded from the machine output. Bedside phase angle measurements were recorded on first treatment day (range 12 days prior‐3 days postinduction) for all patients and, for AML patients, the same day as the nadir bone marrow (occurring on day 11‐14 of induction therapy) on the inpatient ward. PhA values, in degrees (°), were then used to calculate the SPhA (unitless) via the following equation: (SPhA) = (phase angle − phase angle_ref_)/SD_ref_, where SD_ref_ and phase angle_ref_ are from the sex‐, age‐, and BMI‐specific reference values from a healthy population.^31^ Repeated measurements were always done on the same side as the first measurement. Height and weight were measured prior to each PhA measurement and were used to calculate BMI. All measurements were taken using the Quantum IV Bioelectrical Impedance Analyzer (RJL Systems). To dichotomize the SPhA, we considered multiple cutoffs: 25th percentile (Q1) for our study (SPhA ≤ −0.948 vs >−0.948), SPhA < −1.65 vs ≥−1.65, and phase angle <5th reference percentile (by age, BMI, and gender) vs phase angle ≥5th reference percentile, based on previous reports.^28^, ^32^, ^33^ The final cutoff was chosen by which Kaplan‐Meier model for OS had the lowest Akaike information criterion (AIC) for goodness of fit.

#### 2.2.2 Covariates

Demographic (age, gender, and race/ethnicity), laboratory data and comorbid conditions, assessed by the hematopoietic stem cell transplant comorbidity index (HCT‐CI), at admission, and treatment data were collected from the electronic medical record. Tumor‐specific variables with prognostic significance were collected at baseline, including lactate dehydrogenase level, white blood cell count, creatinine, and cytogenetic risk group from diagnostic bone marrow biopsy according to classification detailed by the Southwest Oncology Group.^34^, ^35^ Of note, albumin and blood urea nitrogen (BUN) values were collected at date of first and second PhA measurements rather than at admission.

#### 2.2.3 Outcomes

The primary outcome of this study was 60‐day mortality rate defined as the percent of patients no longer alive at 60 days after first phase angle measurement. A sample size of 102 was chosen as it would give 80% power to detect an odds ratio (OR) of 2.1, using a logistic regression model with a two‐sided 0.05 alpha level and a 60‐day mortality rate of 20%. Secondary outcomes included OS, 30‐day mortality rate, transfer to intensive care unit (ICU) during induction, nadir bone marrow response, complete remission (CR), and length of hospital stay (LHS). Overall survival was measured from date on study to either death or last follow‐up in censored patients in accordance with 2010 ELN recommendations.^9^ Nadir bone marrow response was defined as follows: hypoplastic marrow with <20% cellularity and <5% blasts. In our study, the CR outcome included CR and CRi and was thus defined as follows: bone marrow blasts <5%, absence of blasts with Auer rods, lack of extramedullary disease, and independence of red cell transfusions with recovery of either absolute neutrophil count ≥1.0 × 10^9^/L (1000/µL) or platelet count ≥100 × 10^9^/L (100 000/µL) or both.^9^

### 2.3 Statistical analyses

Means, standard deviations, and frequencies were used to describe baseline patient characteristics including demographics, comorbidity data, laboratories, cytogenetic risk group, and treatments. Proportions for response data were estimated for all patients. Baseline SPhA was dichotomized. *t* Tests were used to assess differences in continuous baseline patient characteristics between the two SPhA categories. To assess differences in categorical variables between the two SPhA groups, chi‐square test or Fisher's exact tests were used. All outcomes were analyzed by baseline SPhA category and for continuous change in SPhA (SPhA at nadir bone marrow SPhA at day 1 of induction treatment) in univariable analyses and in adjusted models for the variables significant for TRM in the model by Kantarjian et al: age, creatinine (>1.3 mg/dL), and cytogenetic risk group.^5^ All categorical outcomes (60‐day mortality, 30‐day mortality, transfer to ICU, nadir bone marrow response, and CR) were modeled using logistic regression. Time‐to‐event analyses (OS and LHS) were conducted using the Kaplan‐Meier method and Cox proportional hazards models (multivariable). Differences in Kaplan‐Meier curves were assessed by log‐rank test. For all analyses, only complete data were analyzed. Interactions for age and SPhA were tested for all models and were included in the model if they fit the *P* < .05 threshold. Subgroup analysis by age (≥60 years), gender, and leukemia type (AML) was conducted for all outcomes in unadjusted and adjusted models. In an exploratory analysis, Pearson's correlations were used to assess the association between change in SPhA and continuous determinants of the value (change in BUN, change in albumin, and change in weight). Finally, multiple linear regression was used to assess predictors (change BUN, change albumin, change in weight, and nadir marrow response) of change in SPhA. Cox regression was used to model the change in albumin with OS in an unadjusted model. For Cox proportional hazards models, the proportionality assumption was assessed and met by visualization of the graph of each covariate predicting the outcome for categorical variables and the plotting of Schoenfeld residuals for continuous variables. For logistic regression models, the linearity assumption for continuous variables was assessed by the Hosmer‐Lemeshow test and was met. SAS 9.4 software (SAS Institute Inc) was used for statistical analysis using a two‐sided *α*‐level of 0.05.

### 2.4 Data sharing

For original data, please contact tspardee@wakehealth.edu.

## 3 RESULTS

### 3.1 Participants

One hundred and two patients were consented, received PhA measurement at baseline, and completed follow‐up. A total of 100 patients, 88 with AML and 12 with ALL, were included in the analysis. One patient was excluded due to not receiving intensive chemotherapy and the other due to having an outlier SPhA measurement (SPhA > 15). In addition, two cytogenetic test results were missing.

### 3.2 Descriptive data

Of the 100 patients, 88 were AML, mean age was 59 (SD 14.6) years, and 56% were female (Table 1). Only 4% had favorable cytogenetic abnormalities. 35% of the cohort showed significant comorbidity burden (HCT‐CI ≥ 3) at diagnosis. For those with AML, 80% received standard induction therapy with anthracycline and cytarabine (7 + 3) or alternative anthracycline and cytarabine‐based induction regimens. All but two patients with ALL received the induction regimen as per CALGB 10102 (ClinicalTrials.gov Identifier: NCT00061945). Between the two SPhA groups, there were significant differences found for mean age and albumin (Table 1). Standardized phase angle means and SDs at baseline, nadir bone marrow, and change in SPhA measurements were 0.36 (1.99), 0.03 (2.21), and −0.05 (2.04), respectively (Table 2).

**Table 1:** Patient characteristics at baseline

	All patients (n = 100), Mean ± SD, or N (%)	SPhA: Quartile 1 (≤−0.948) (n = 26), Mean ± SD, or N (%)	SPhA: Quartiles 2, 3, and 4 (>−0.948) (n = 74) Mean ± SD, or N (%)	*P* a
Diagnosis				.726
AML	88 (88.0)	24 (92.3)	64 (86.5)	
ALL	12 (12.0)	2 (7.7)	10 (13.5)	
Age	58.9 ± 14.6	65.5 ± 12.1	56.6 ± 14.7	.006
<60 y	43 (43.0)	7 (26.9)	36 (48.7)	.054
≥60 y	57 (57.0)	19 (73.1)	38 (51.4)	
Gender (female)	56 (56.0)	15 (57.7)	41 (55.4)	.840
Race (white)	89 (89.0)	24 (92.3)	65 (87.8)	.724
BMI	29.7 ± 7.1	27.4 ± 7.3	30.6 ± 6.9	.053
Laboratories				
Hemoglobin, g/dL	9.3 ± 2.0	9.1 ± 2.0	9.4 ± 2.1	.489
LDH, U/L	533.2 ± 681.3	467.2 ± 454.1	556.3 ± 746.1	.500
White cell count, 10^9^/L	31.2 ± 62.5	40.6 ± 58.4	27.8 ± 63.9	.358
Creatinine, mg/dL	1.03 ± 0.47	1.08 ± 0.41	1.01 ± 0.50	.479
BUN, mg/dL^b^	15.9 ± 8.5	15.6 ± 8.0	16.0 ± 8.7	.861
Albumin, g/dL^b^	3.3 ± 0.5	3.1 ± 0.5	3.4 ± 0.4	.014
Cytogenetic risk group^c^				.772
Favorable	4 (4.1)	0 (0.0)	4 (5.4)	
Intermediate	54 (55.1)	14 (58.3)	40 (54.1)	
Unfavorable	40 (40.8)	10 (41.7)	30 (40.5)	
HCT‐CI ≥ 3	35 (35)	9 (34.6)	26 (35.1)	.960
Induction therapy				.820
Cytarabine + anthracycline	64 (64.0)	15 (57.7)	49 (66.2)	
As per CALGB 10102	10 (10.0)	2 (7.7)	8 (10.8)	
Cytarabine + anthracycline+midostaurin	4 (4.0)	1 (3.8)	3 (4.1)	
Cytarabine + anthracycline+HiDAC + mitoxantrone	2 (2.0)	0 (0)	2 (2.7)	
Clofarabine	8 (8.0)	4 (15.4)	4 (5.4)	
Other	12 (12.0)	4 (15.4)	8 (10.8)	

Abbreviations: ALL, acute lymphoblastic leukemia; BMI, body mass index; BUN, blood urea nitrogen; HCT‐CI, hematopoietic stem cell transplant comorbidity index; HiDAC, high‐dose cytarabine; LDH, lactate dehydrogenase; SPhA, standardized phase angle.

a Calculated using chi‐square test or Fisher's exact test (if expected n < 5 in any cell of the contingency table) for categorical variables and *t* tests for continuous variables.

b BUN and albumin values were unavailable for 2 subjects.

c Cytogenetic test results were unavailable for 2 subjects. Favorable and intermediate were combined to form a single variable for significance testing compared to unfavorable due to the lack of patients with favorable cytogenetics.

**Table 2:** Baseline, nadir bone marrow, and change in phase angle measurements

	Mean ± SD	Min, 5th, 25th, 50th, 75th, 95th Max
Baseline in total population (n = 100)		
Phase angle (°)	6.07 ± 1.67	2.60, 3.70, 5.00, 5.90, 7.20, 9.55, 10.20
SPhA	0.36 ± 1.99	‐3.26, −2.37, −0.95, 0.02, 1.53, 4.49, 5.62
Baseline for those who received nadir marrow (n = 68)		
Phase angle ^(o)^	5.84 ± 1.44	3.10, 3.80, 4.95, 5.70, 6.75, 8.40 9.60
SPhA	0.08 ± 1.72	‐3.26, −2.19, −0.95, −0.30, 1.08, 3.01, 4.46
SPhA at nadir bone marrow (n = 68)		
Phase angle (°)	5.83 ± 1.74	2.40, 3.30, 4.70, 5.50, 6.75, 8.90, 10.80
SPhA	0.01 ± 2.19	‐4.11, −2.89, −1.55, −0.30, 1.12, 4.08 6.28
Change in SPhA (n = 68)	−0.05 ± 2.04	‐4.14,‐3.03, −1.03, −0.25, 0.66, 4.35, 8.16

Sixty‐eight of the 84 (81%) patients eligible for second PhA measurement underwent the measurement. Of the 16 patients who did not receive a second PhA measurement, 9 of those were due to reasons where the patients were particularly ill (ICU machines leading to interference with measurement (3), patient with *Clostridium difficile* infection (4), study team felt it inappropriate to gather measurement while patient was contemplating hospice after their nadir bone marrow showed poor response (Table S1)).

### 3.3 Outcome data

**Figure 1:**
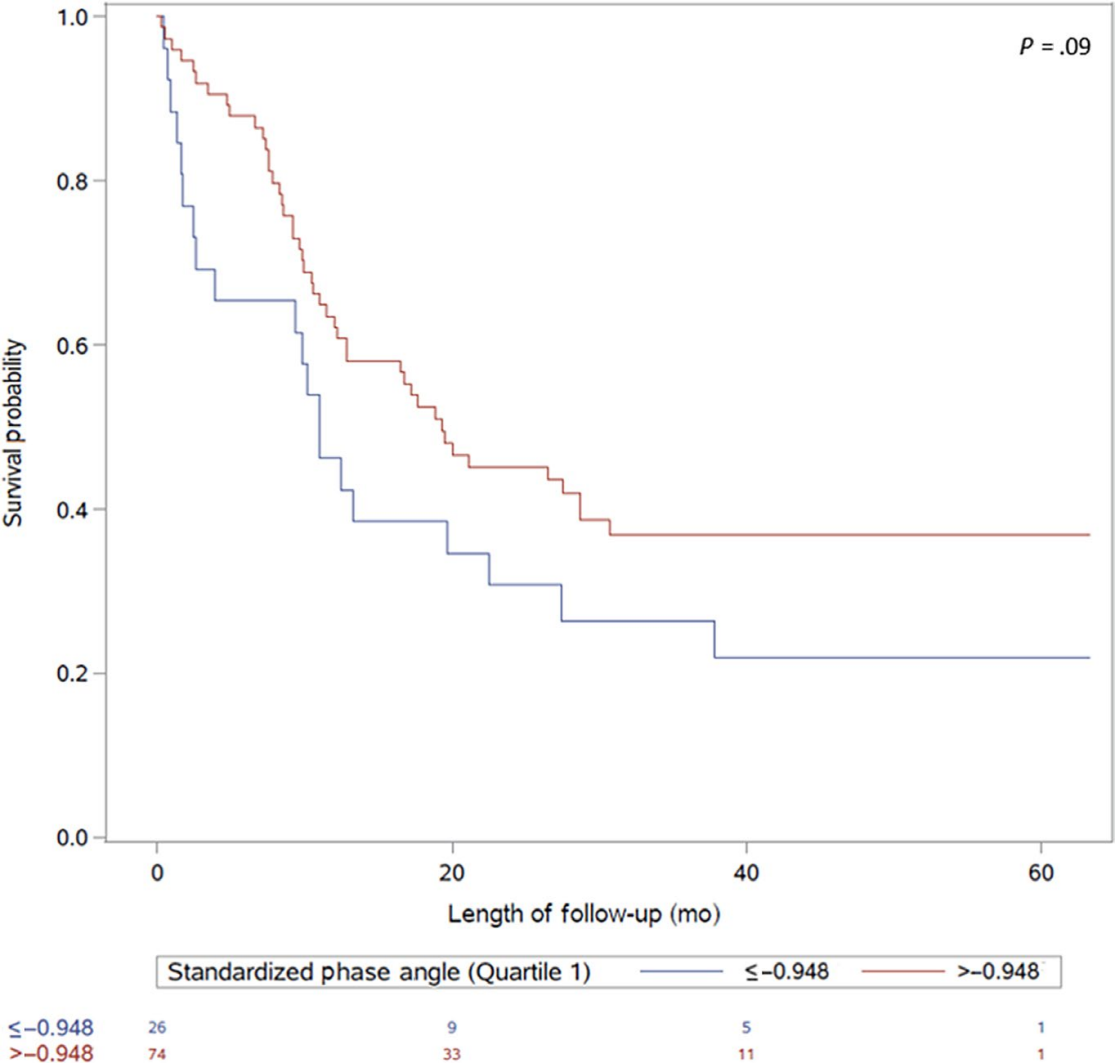
Survival by log‐rank test of 100 AML and ALL patients by standardized phase angle (SPhA). Quartile 1 (≤−0.948) compared to Quartiles 2‐4 (>−0.948) baseline SPhA

**Figure 2:**
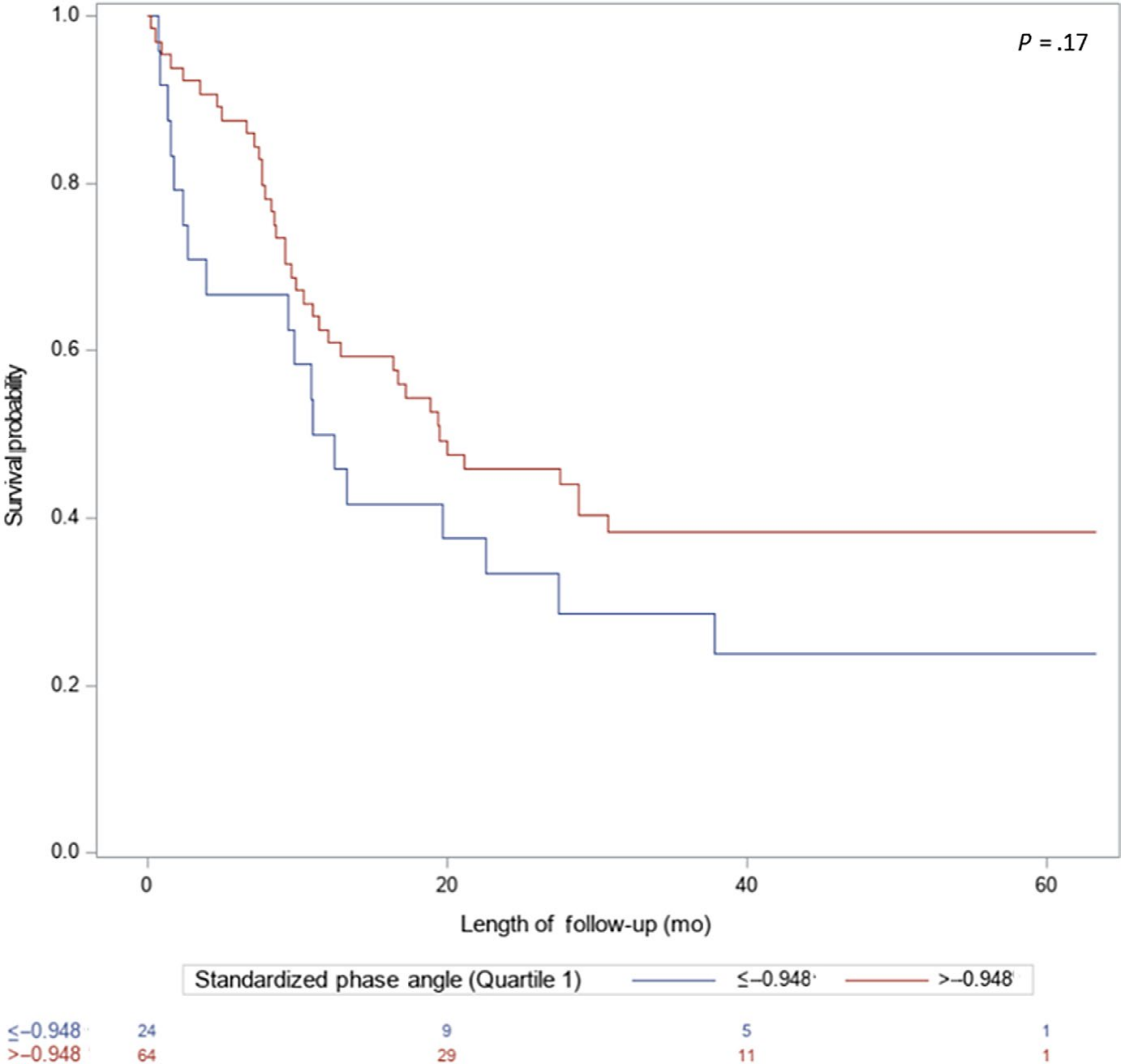
Survival by log‐rank test of 88 AML patients by standardized phase angle (SPhA). Quartile 1 (≤−0.948) compared to Quartiles 2‐4 (>−0.948) baseline SPhA

Outcome data for CR, nadir marrow response, 30‐ and 60‐day mortality, and requirement of ICU stay by overall cohort and AML subgroup are shown in Table 3. SPhA was used in a dichotomized form using the cutoff of 25th percentile (−0.948) due to it having the lowest AIC (529.37) compared to SPhA = −1.65 (AIC = 531.48) and phase angle <5th reference percentile (by age, BMI, and gender) (AIC = 530.80). No interactions between age and SPhA were significant and accordingly not included in any models. Sixty‐day mortality was greater among those in the lowest quartile of SPhA, with 23% in the lowest quartile dying within 60 days compared to 5% of those above the lowest quartile (OR = 5.25, 95% CI, 1.35, 20.44) although after adjustment for age, cytogenetic risk group, and creatinine category, those with SPhA ≤−0.948 compared with those with SPhA >−0.948 had 3.12 times the odds of death within 60 days of start of induction (*P* = .15) (Tables 3 and 4). Median OS was lower in the lowest SPhA group (SPHA ≤ −0.948: median OS = 11.0 months vs SPHA>−0.948: median OS = 19.5 months; *P* = .09) (Figure 1), though the difference was not statistically significant. Similar results were found in the AML subgroup analysis (Table 4; Figure 2). No association was found between baseline SPhA 30‐day mortality, requirement of ICU stay, achievement of CR, nadir marrow response, and LHS (Tables 3 and 4). Subgroup analyses by gender and age ≥60 years can be found in the Supplementary Materials (Tables S2 and S3).

**Table 3:** Outcome data

Response N(%)	All patients (n = 100)^a^	SPhA: Quartile 1 ≤−0.948 (n = 26)	SPhA: Quartiles 2, 3, and 4>−0.948 (n = 74)	*P* b	Diagnosis: AML (n = 88)	SPhA: Quartile 1 ≤−0.948 (n = 24)	SPhA: Quartiles 2, 3, and 4>−0.948 (n = 64)	*P* b
Complete remission achieved	80 (80.0)	19 (73.1)	61 (82.4)	.3	69 (78.4)	18 (75.0)	51 (79.7)	.63
Nadir marrow response	N/A	N/A	N/A	N/A	46 (53.5)	13 (56.5)	33 (52.4)	.73
30‐d mortality	6 (6.0)	3 (11.5)	3 (4.1)	.18	5 (5.7)	2 (8.3)	3 (4.7)	.61
60‐d mortality	10 (10.0)	6 (23.1)	4 (5.4)	.02	9 (10.2)	5 (20.8)	4 (6.3)	.06
Required ICU stay	10 (10.0)	5 (19.2)	5 (6.8)	.12	9 (10.2)	4 (16.7)	5 (7.8)	.25

Note

All response for event YES.

N/A: The overall measure is the same as the AML measure as ALL patients do not receive nadir marrows.

Abbreviations: ICU, intensive care unit; SPhA, standardized phase angle.

a Nadir marrow response results were not recorded for ALL patients and were missing in 2 AML patients. N = 23 and N = 63 for ≤‐0.948 and> −0.948, respectively.

b Analyses conducted using chi‐square or Fisher's exact test.

**Table 4:** Models for OS, LHS, 60‐day mortality, complete remission, nadir marrow response, and requirement of ICU stay by baseline SPhA as predictor

Hazard ratio (95% CI)		
Model	Overall unadjusted (n = 100) adjusted (n = 98)	AML unadjusted (n = 88) adjusted (n = 87)
OS		
Unadjusted	1.57 (0.93, 2.66) *P* = .09	1.46 (0.84, 2.55) *P* = .18
Adjusted	1.23 (0.71, 2.15) *P* = .46	1.24 (0.69, 2.23) *P* = .46
LHS		
Unadjusted	0.86 (0.53, 1.38) *P* = .52	0.89 (0.55, 1.46) *P* = .64
Adjusted	0.95 (0.57, 1.57) *P* = .84	0.94 (0.56, 1.61) *P* = .83
Odds ratio (95% CI)		
60‐day mortality		
Unadjusted	5.25 (1.35, 20.44) *P* = .02	3.95 (0.96, 16.21) *P* = .06
Adjusted	3.12 (0.67, 14.48) *P* = .15	3.17 (0.66. 15.21) *P* = .15
Complete remission achieved		
Unadjusted	0.58 (0.20, 1.66) *P* = .31	0.77 (0.25, 2.31) *P* = .63
Adjusted	0.81 (0.26, 2.66) *P* = .72	0.89 (0.28, 2.85) *P* = .84
Nadir marrow response achieved^a^		
Unadjusted	N/A	0.85 (0.32, 2.21) *P* = .73
Adjusted	N/A	0.92 (0.33, 2.58) *P* = .88
Required ICU stay		
Unadjusted	3.29 (0.87, 12.45) *P* = .08	2.36 (0.58, 9.66) *P* = .23
Adjusted	2.88 (0.64, 13.02) *P* = .17	2.78 (0.61, 12.63) *P* = .19

Note

Adjusted model includes age, cytogenetic risk group, and creatinine.

All estimates are for Quartile 1 (≤−0.948) compared to Quartiles 2‐4 (>−0.948) baseline SPhA.

N/A: The overall measure is the same as the AML measure as ALL patients do not receive nadir marrows.

Abbreviations: ICU, intensive care unit; SPhA, standardized phase angle.

a Nadir marrow response results were not recorded for ALL patients and were missing in 2 AML patients. N = 23 and N = 63 for ≤−0.948 and >−0.948, respectively.

Change in SPhA was significantly associated with OS but not LHS or 60‐day mortality. Specifically, when adjusted for cytogenetic risk group, age, and creatinine category, for every 1‐unit increase in SPhA from day 1 of induction to nadir bone marrow there was an associated 15% increased risk of death during the two‐year follow‐up period (hazard ratio (HR) = 1.15; 1.00‐1.33 *P* = .05) (Table 5). Analyses for change in SPhA for ICU stay, achievement of CR, and nadir marrow response can be found in Supplementary Materials (Table S4).

**Table 5:** Models for OS, LHS, and 60‐day mortality by change in SPhA as predictor

Hazard ratio (95% CI)		
Model		Overall unadjusted (n = 68) adjusted (n = 67)
OS		
Unadjusted		1.15 (1.01, 1.31) *P* = .03
Adjusted		1.15 (1.00, 1.33) *P* = .05
LHS		
Unadjusted		0.99 (0.85, 1.14) *P* = .83
Adjusted		0.97 (0.83, 1.13) *P* = .69
Odds ratio (95% CI)		
60‐day mortality		
Unadjusted		1.26 (0.86, 1.85) *P* = .23
Adjusted		1.18 (0.75, 1.85) *P* = .47

Note

Adjusted model includes age, cytogenetic risk group, and creatinine.

Abbreviation: SPhA, standardized phase angle.

Due to the direction of the association being the opposite of baseline SPhA, where higher values are associated with better prognosis, the exploratory analysis to assess predictors of change SPhA was conducted. In exploratory analyses (Table 6), change in albumin and nadir marrow response were the strongest predictors of change in SPhA. Specifically, change in albumin was positively correlated (*r* = .20; *P* = .10) with change in SPhA and associated with a 0.75‐unit increase in change in SPhA per 1 g/dL increase in albumin when adjusted for change in BUN, weight, and nadir marrow response (*P* = .20). In those who did not respond on nadir marrow, compared to those who did respond, there was an associated 0.82‐unit increase in change SPhA in the adjusted model (*P* = .1). Furthermore, though not significant, for every 1 g/dL increase in albumin from day 1 of induction to nadir bone marrow there was a 25% decreased risk of death within the 2‐year follow‐up period (HR = 0.75; 95% CI: 0.40, 1.40; *P* = .37).

**Table 6:** Pearson's correlation and multiple linear regression results for determinants of change in SPhA

Predictor	Correlation coefficient (*r*)	*P* value	Coefficient for multiple linear regression^a^	*P* value
ΔBUN, mg/dL	−.11	.37	−0.02	.43
ΔWeight, kg	−.09	.47	−0.001	.97
ΔAlbumin, g/dL	.20	.10	0.75	.20
Nadir marrow response (No)^b^	N/A	N/A	0.82	.10

Abbreviation: SPhA, standardized phase angle.

a Each variable adjusted for other variables listed in Table 6.

b Estimate compares those who did not respond (No) to their nadir marrow to those who did (Yes) respond.

## 4 DISCUSSION

To the best of our knowledge, this study is the first to assess and establish the prognostic significance of PhA technology in newly diagnosed adults with acute leukemia undergoing intensive chemotherapy. In our cohort, we found increased 60‐day mortality for patients in the lower 25th percentile of baseline SPhA though this association was significant only in univariable analysis. We also found change in SPhA to be a significant predictor of OS even when adjusted for age, cytogenetic risk group, and creatinine.

Our study adds to the growing body of literature showing the prognostic significance of SPhA in oncology and HSCT patients.^26^, ^28^, ^36^ For example, in a study of 195 newly diagnosed mixed cancer patients the mortality rate was higher in patients with baseline SPhA< −1.65 compared to SPhA ≥−1.65.^32^ In a prospective study of 105 HSCT patients with hematological malignancies' multivariable Cox regression including age, age‐ and gender‐adjusted BMI, SPhA, CRP, remission status, donor status, KPS score, and CMV, they found only SPhA, HLA‐C incompatibility, and unrelated donor were independently prognostic for 2‐year OS. Furthermore, median OS, relapse mortality, and progression‐free survival all showed significant differences in SPhA.^28^ Similarly, in another prospective study of HSCT patients SPhA was prognostic for 90‐day mortality.^36^

The values of SPhA at diagnosis in our study are higher than those found in previous literature utilizing SPhA in an oncology setting. A study of adult mixed solid tumor patients scheduled to undergo surgical treatment found a mean of −0.87 ± 1.43, and 28.1% of their population was SPhA <−1.65.^37^ Similarly, in a study of mixed solid tumor patients about to undergo radiation therapy 27% of patients presented with SPhA <−1.65.^38^ Finally, in the study of HSCT patients mentioned previously the mean was −1.31 ± 1.25 and 25th percentile = −2.26.

Our pilot study found a significant association in univariable but not multivariable logistic regression between our primary outcome, 60‐day mortality, and baseline SPhA. However, our multivariable effect size was clinically relevant (OR = 3.12). Furthermore, our study was underpowered due to only 10 deaths occurring compared to the expected 20. Thus, we expect a follow‐up study with increased sample size is warranted.

Our study is also the first to assess the prognostic significance of change in SPhA for mortality outcomes in oncology patients. We found change in SPhA to be a significant predictor of mortality with an increase in SPhA from day 1 of induction to nadir bone marrow predicting increased mortality in AML patients. Surprisingly, patients who had higher nadir SPhA relative to baseline had a worse prognosis. In an exploratory analysis though no predictors were significant, nadir marrow response and change in albumin showed the strongest effects. In those with residual disease on nadir marrow, there was a 0.82‐unit increase in change in SPhA, and for every 1 g/dL decrease in albumin from day 1 of induction to nadir bone marrow, there was a 0.75‐unit decrease in change SPhA when controlling for change in BUN, change in weight, and nadir marrow response. Furthermore, though not significant, for every 1g/dL decrease in albumin from day 1 of induction to nadir bone marrow there was a 25% increased risk of death within the 2‐year follow‐up period (HR = 1.25; 95% CI: 0.40, 1.40; *P* = .37). These exploratory analyses suggest the direction of the change in SPhA effect on OS is due to a combination of disease response and nutrition status. There are notable limitations to our interpretations regarding change in SPhA. First, whether phase angle technology has the ability to assess the burden of leukemia in patient's bone marrow is not known. Furthermore, change in SPhA did not predict CR status in univariable or multivariable analysis which would have been expected if the change in SPhA value was a surrogate marker for marrow response as the predictive ability of nadir marrow response for CR is well‐documented.^39^ Future studies need to be conducted to specifically assess the question of content validity of change in SPhA.

A strength of our study is the prospective nature as most phase angle studies to this point have been retrospective or cross‐sectional in nature.^36^, ^38^ Furthermore, the utilization of SPhA increases generalizability as the effects of BMI, age, and gender are theoretically removed from the values allowing for the comparison of disease effects on the measure across different studies. A limitation of our study is the utilization of the lower quartile (25th percentile) SPhA, reducing the external validity of our results, rather than a previously published, validated cutoff value. However, this cutoff has been shown to be prognostically relevant^28^ and was chosen by a validated goodness‐of‐fit criteria (AIC) in comparison with those cutoffs in previous studies.^32^, ^33^ A further limitation includes the lack of collection of performance status and inflammation parameters. Another limitation to consider in our results concerns selection bias in the change in SPhA results. This bias may have shifted our HR and OR estimates toward or away from the null hypothesis of no association between change in SPhA and outcome, but we are unable to further infer on the effect of the bias. Lastly, a limitation of PhA technology is a lack of an exact understanding of the physiologic meaning of PhA. Though many authors claim that phase angle is validated as a nutritional status marker,^26^ in a recent review focused on assessing the association between PhA and malnutrition in adults the authors stated that though many studies find a correlation between PhA and nutritional status, low PhA cannot specifically reflect impaired nutritional status, particularly in patients with inflammatory processes where the associated overhydration may lower PhA more than explained by their nutritional status. A better understanding of the content validity of PhA would allow for stronger evidence to pursue interventions based on the information provided by the measure. Specifically, promising work has been done to determine the effects of interventions, chiefly resistance training and nutritional support, to minimize sarcopenia and increase muscle function in patients with low PhA.^40^, ^41^, ^42^

In conclusion, our findings suggest that in newly diagnosed acute leukemia patients undergoing intensive induction chemotherapy, SPhA technology may provide important prognostic information for TRM and OS. PhA is an objective, repeatable, high‐precision measure in acute leukemia patients and, unlike other prognostic factors in this population, is potentially subject to intervention. Future studies of PhA technology in leukemia patients should address the content validity of phase angle while further exploring the promising findings of the effect of strength training and nutritional supplementation on PhA and patient outcomes.

## CONFLICT OF INTEREST

The authors declare no competing financial interests.

## AUTHOR CONTRIBUTION

SJY wrote the manuscript, aided in data collection, interpreted data, and performed analyses; SL, MM, and SD collected data; JAT performed analyses, interpreted data, and assisted in manuscript writing; HDK interpreted data and assisted in manuscript writing; BLP assisted in manuscript writing; AU assisted in data collection; and TSP designed the study, interpreted data, and assisted in manuscript writing.
