# Supplementary material for: The prognostic value of standardized phase angle in adults with acute leukemia: A prospective study
Source: Cancer Med. 2020 Feb 12;9(7):2403–13. doi: 10.1002/cam4.2835 (PMC7131844; doi:10.1002/cam4.2835)
Supplement: Supplementary file 1 [file CAM4-9-2403-s001.docx]

Supplementary Materials:

**Table 1** Reasons for non-compliance with second phase angle measurement.

| Reason for missed 2^nd^ phase angle | Count (n=16) |
| --- | --- |
| Interference with measurement due to machine in ICU. | 3 |
| Patient declined | 1 |
| C. difficile infection (threat of contamination of phase angle device) | 4 |
| Patient had marrow poor response and study team felt it inappropriate to ask for measurement to be recorded while family and patient was contemplating hospice. | 2 |
| Data collector was on vacation. | 1 |
| Patient received pacemaker between first and second measurement which is a contraindication to taking measurement. | 1 |
| Reason not recorded | 4 |

**Table 2** Logistic regression models for 60-day mortality, complete remission, nadir marrow response, and requirement of ICU stay predicted by SPhA in subgroup analyses by age and gender.

| Odds ratio (95% CI) | | | |
| --- | --- | --- | --- |
| Model | Age≥60 years  N=56 | Females  N=55 | Males  N=43 |
| 60-day mortality | 3.98 (0.78, 20.36) p=0.10 | 2.33 (0.24, 22.31)  P=0.46 | 1.34 (0.010, 18.77)  P=0.83 |
| Complete remission achieved | 0.48 (0.13, 1.78)  P=0.27 | 0.40 (0.08, 1.98)  P=0.26 | N/A^*^ |
| Nadir marrow response achieved^ꝉ^ | 0.45 (0.12, 1.72)  P=0.24 | 0.25 (0.04, 1.66)  P=0.15 | 2.47 (0.54, 11.20)  P=0.24 |
| Required ICU stay | 3.86 (0.55, 26.93)  P=0.17 | 2.27 (0.22, 23.60)  P=0.49 | N/A^*^ |

Adjusted model includes age, cytogenetic risk group, and creatinine.

All estimates are for Quartile 1 (≤-0.948) compared to Quartiles 2-4 (>-0.948) baseline SPhA.

^*^Model could not be fit due to Quasi-Complete separation.

ꝉ14-Day Marrow Response results were not recorded for ALL patients and were missing in 2 AML patients. N=23 and N=63 for ≤-0.948 and > -0.948, respectively. Number of observations for age (n=49) subgroup analysis, male (n=40) subgroup analysis, and female (n=44) subgroup analysis.

**Table 3**. Cox Proportional Hazards models for OS and LHS predicted by SPhA in age and gender subgroups.

| Hazard ratio (95% CI) | | | |
| --- | --- | --- | --- |
| Model | Age≥60 years  N=56 | Females  N=55 | Males  N=43 |
| OS | 1.47 (0.78, 2.79)  P=0.23 | 1.36 (0.63, 3.00)  P=0.43 | 0.90 (0.38, 2.17)  P=0.82 |
| LHS | 0.78 (0.42, 1.43) p=0.42 | 0.77 (0.37, 1.59) p=0.47 | 1.36 (0.61, 3.05)  P=0.45 |

Adjusted model includes age, cytogenetic risk group, and creatinine.

All estimates are for Quartile 1 (≤-0.948) compared to Quartiles 2-4 (>-0.948) baseline SPhA.

**Table 4** Models for complete remission, nadir marrow response, and requirement of ICU stay by change in SPhA as predictor.

| Odds ratio (95% CI) | | |
| --- | --- | --- |
| Model |  | Overall  (n=67) |
| Complete remission achieved | Unadjusted | 0.91 (0.67, 1.23) P=0.54 |
|  | Adjusted | 0.90 (0.64, 1.26) P=0.54 |
| Nadir marrow response achieved | Unadjusted | 0.79 (0.60, 1.04) P=0.09 |
|  | Adjusted | 0.80 (0.59, 1.07) P=0.13 |
| Required ICU stay | Unadjusted | 1.16 (0.80, 1.69) P=0.44 |
|  | Adjusted | 1.12 (0.74, 1.69) P=0.61 |

Adjusted model includes age, cytogenetic risk group, and creatinine.
